# Skeletal muscle mass and overhydration are associated with in-hospital mortality in acute heart failure patients

**DOI:** 10.1590/1806-9282.20250908

**Published:** 2026-05-01

**Authors:** Yunuen Reyes-Paz, Lilia Castillo-Martínez, José Rubén García-Sánchez, Fernanda Bernal-Ceballos, José Luis Villanueva-Juárez, Thierry Hernández-Gilsoul

**Affiliations:** 1 Instituto Politécnico Nacional, Escuela Superior de Medicina, Sección de Estudios Posgrado e Investigación – Mexico City, México.; 2 Universidad Nacional Autónoma de México, Facultad de Estudios Superiores Zaragoza – Mexico City, México.; 3 Instituto Nacional de Ciencias Médicas y Nutrición Salvador Zubirán, Clinical Nutrition Service – Mexico City, Mexico.; 4 Instituto Nacional de Ciencias Médicas y Nutrición Salvador Zubirán, Critical Care Division – Mexico City, México.

**Keywords:** Heart failure, Fluid overload, Muscular atrophy, Tomography, Prognosis

## Abstract

**OBJECTIVE:**

The aim of the study was to determine the association between low muscle mass and abnormal fluid distribution with in-hospital mortality in patients with acute heart failure.

**METHODS:**

In a prospective cohort study at the Instituto Nacional de Ciencias Médicas y Nutrición Salvador Zubiran in Mexico City, patients with acute heart failure who underwent thoracic or abdominal computed tomography within 72 h before or after admission between September 2017 and July 2024 were included. The exclusion criteria were an illegible computed tomography scan, an incorrect bioimpedance analysis lecture, the presence of cancer, COVID-19, chronic kidney disease with renal replacement therapy, or dismissal of a diagnosis of acute heart failure. Bioelectrical impedance analysis was performed within the first 24 h of hospitalization to measure phase angle and impedance ratio for evaluating hydration status. The skeletal muscle area was measured using a single axial slide at L3 for abdominal computed tomography and the T4 level for thoracic computed tomography.

**RESULTS:**

This study included 134 patients, with an overall hospital survival rate of 83.6%. The mortality group had a lower abdominal skeletal muscle area (86.5 vs. 111 cm2, p=0.024), smaller pectoral skeletal muscle area (18.9 vs. 26 cm2, p=0.005), lower phase angle (3 vs. 3.9, p=0.010), increased impedance ratio (0.89 vs. 0.86, p=0.002), greater prevalence of reduced pectoral muscle mass (40.9 vs. 12.5%, p<0.001), and abnormal fluid distribution according to impedance ratio (86.4 vs. 57.1%, p=0.016). In survival analyses, the interaction effect of pectoral skeletal muscle area and increased impedance ratio had the lowest survival probability (log-rank test, p<0.001).

**CONCLUSION:**

Low skeletal muscle mass and abnormal fluid distribution are associated with in-hospital mortality in patients with acute heart failure.

## INTRODUCTION

Acute heart failure (AHF) is a common cause of hospitalization and is characterized by symptoms of rapid-onset heart failure (HF), with congestion being the main issue^1^. Congestion can be intravascular, tissular, or both^2^. Therefore, accurate identification of congestion is crucial in AHF management^1–3^; congestion is classified into phenotypes based on the underlying mechanism (fluid redistribution or retention) and the affected compartments (tissular or vascular). Although phenotypes overlap, identifying the primary form of congestion enables effective decongestion therapy, reduces residual congestion, and improves outcomes^1,4^. However, objective indicators for congestion characterization are limited in clinical practice^3^. Bioelectrical impedance analysis (BIA) is a reliable and noninvasive method for assessing hydration status. The impedance index and bioimpedance vector analysis assist in evaluating congestion, which is correlated with HF-related clinical outcomes such as length of stay and mortality^3,5^.

The continuous loss of muscle mass in patients with HF can stem from low nutritional absorption, immobility, and inflammation^6,7^. Muscle mass loss is associated with an increased risk of negative outcomes in patients with HF^8^. Measurement of the axial muscle mass area using computed tomography (CT) has been proposed as a surrogate for skeletal mass, given its good correlation, as it is associated with various clinical outcomes and mortality in cardiac conditions, including acute and chronic HF, aortic valve surgery, and ventricular assist device implantation^9,10^, and hinders AHF compensatory mechanisms and worsens congestion^11^. Thus, the aim of this study was to determine the association of low muscle mass and abnormal fluid distribution with in-hospital mortality among patients with AHF.

## METHODS

Consecutive patients aged ≥18 years admitted to the emergency department (ED) of the Instituto Nacional de Ciencias Médicas y Nutrición Salvador Zubiran (INCMNSZ) between September 2017 and July 2024 with a diagnosis of AHF according to the European Society of Cardiology guidelines^12^, considering the presence of HF signs or symptoms (breathlessness, peripheral edema, rales, third heart sound, etc.), a brain natriuretic peptide (BNP) level ≥100 pg/mL, and confirmation through echocardiography, were selected for this study. The exclusion criteria were not having a CT within 72 h before or after admission, the presence of a metallic cardiac device that obstructs the visibility of the muscles in the CT, an incorrect BIA lecture, the presence of cancer, COVID-19, chronic kidney disease with renal replacement therapy, or the dismissal of a diagnosis of AHF. The outcome was defined as in-hospital death, and the follow-up period was defined as the time of arrival at the ED until discharge or death.

Demographic, clinical, and laboratory variables were retrieved from electronic medical records. BIA was performed during the first postadmission 24-h period, adhering to a previously described methodology^13^, using Body QuadScan 4000 equipment (Bodystat Ltd., Douglas, Isle of Man, UK). Multifrequency equipment that reports the resistance (at 5, 50, 100, and 200 kHz), reactance, and phase angle (PhA). The impedance ratio (Imp-R) is the ratio between the resistances at 200 and 5 kHz frequencies, for which a cutoff point of ≥0.85 was considered indicative of an abnormal fluid distribution^5^. Both PhA and Imp-R are related to fluid alterations, diminished nutritional status, and worse clinical outcomes^14,15^.

Muscle mass was assessed using RadiAnt DICOM software version 5.5, and the muscles were manually shaded using the free-stroke ROI (freehand drawing tool within the region of interest) function to obtain the cross-sectional area (cm^2^). The measurements were performed on a single axial slice, at either the point just above the aortic arc for thoracic CT or at the level of L3 with both pedicles visible on abdominal CT. At the thoracic level, the areas of the pectoralis major and minor muscles were assessed bilaterally to determine the pectoralis skeletal muscle area (SMA), which was adjusted by the square height to obtain the skeletal muscle index (SMI). A cutoff point of <19.25 cm^2^/m^2^ was used to define low muscle mass^16^. When evaluating abdominal CT, the psoas, rectus abdominis, oblique, paraspinal, and lumbar squares were used to obtain SMA, SMI, and low muscle mass classifications, with a cutoff point of 41.6 cm^2^/m^2^ for men and 32 cm^2^/m^2^ for women^17^.

The BIA and CT assessments were performed and analyzed by the same trained researcher to ensure methodological consistency. Intra-operator test–retest repeatability was assessed in a subsample of 21 participants; the results per muscle are presented in the Supplementary Table 1.

The statistical analysis was performed with R via Jamovi (version 2.2.5). The normality of the data was determined using the Shapiro-Wilk test. Comparisons between groups based on mortality were performed using the Mann-Whitney U test for quantitative variables and the chi-square test for qualitative variables, with a p<0.05 considered statistically significant. Logistic regression was performed to analyze the associations between variables that were significantly different between the groups; variables with a p-value of 0.2 in the univariate analyses were considered candidates for the multivariate model. The best model fit was selected according to the Akaike information criterion (AIC), parsimony, and collinearity principles. Kaplan-Meier analysis was performed to compare survival between groups, and significant differences were determined using the log-rank test.

This study received approval from the INCMNSZ Ethics Committee and the Human Biomedical Research Committee. All the participants agreed to participate and signed an informed consent form.

## RESULTS

**Figure 1:**
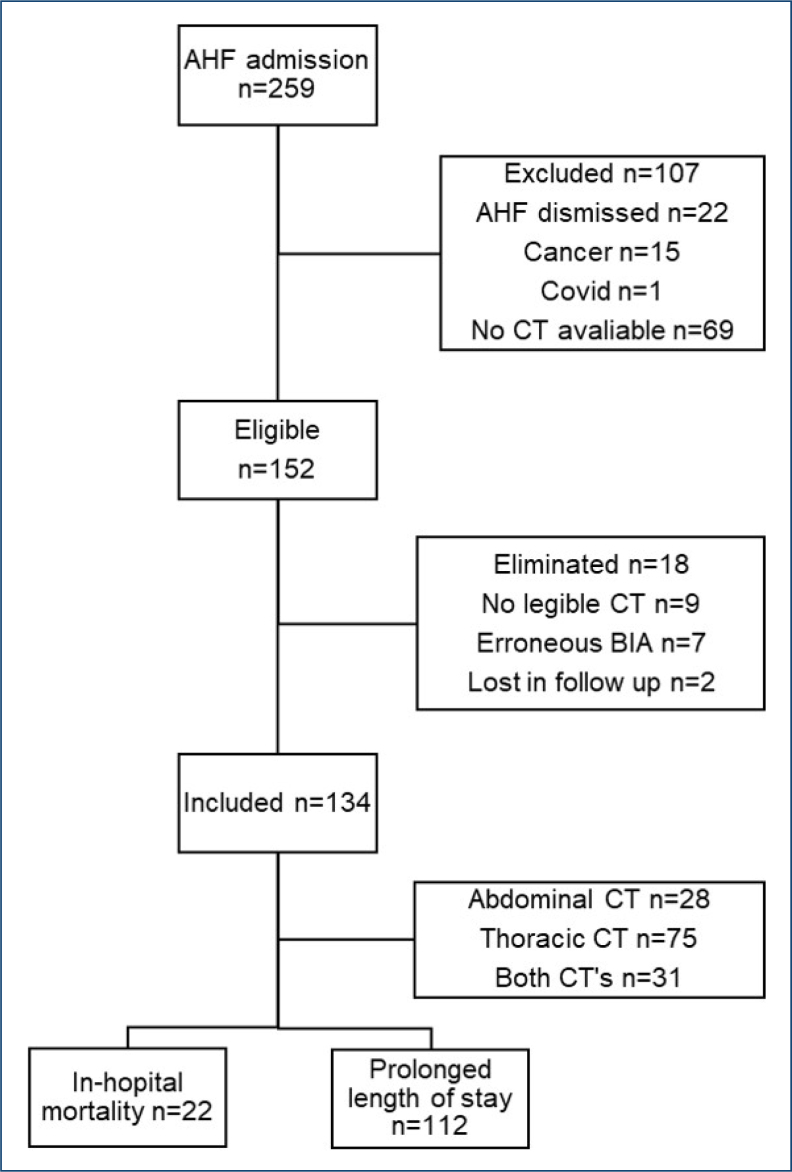
Flow chart of patients included in the study. AHF: acute heart failure; CT: computed tomography; BIA: bioelectrical impedance analysis.

Among the 259 patients with AHF admitted to the ED, 152 were eligible for inclusion; of these, 18 were eliminated, and 134 were included in the analysis (Figure 1). The clinical characteristics of the patients are summarized in Table 1. The median age of the patients was 70 years, and the majority were female. Most patients presented with New York Heart Association (NYHA) functional class II or III and were classified as HF with preserved ejection fraction.

**Table 1:** Clinical characteristics of the total sample and comparison of in-hospital mortality patients.

Variables	Total (n=134)	Mortality (n=22)	Non-mortality (n=112)	p-value
Female, n (%)	88 (65.7)	15 (68.2)	73 (65.2)	0.786
Age, years	70.5 (60.5–80)	69 (58.5–81.8)	70.5 (62–80)	0.803
BMI, kg/m^2^	26 (22.8–31.2)	23 (20.8–28)	27.4 (23.7–31.9)	0.011
Charlson Index	5 (4–6)	5 (4–7)	5 (4–6)	0.427
Comorbidities, n (%)				
Hypertension	92 (68.7)	15 (68.2)	77 (68.8)	0.958
Diabetes mellitus	57 (42.5)	10 (45.5)	47 (42)	0.762
CKD	37 (27.6)	6 (27.3)	31 (27.7)	0.969
Dyslipidemia	34 (25.4)	6 (27.3)	28 (25)	0.823
Hypothyroidism	30 (22.4)	6 (27.3)	24 (21.4)	0.548
Liver cirrhosis	12 (9)	5 (22.7)	7 (6.3)	0.013
MASH	3 (2.2)	1 (4.5)	2 (1.8)	0.424
Lupus	15 (11.2)	1 (4.5)	14 (12.5)	0.279
Rheumatoid arthritis	7 (5.2)	1 (4.5)	6 (5.4)	0.876
Pneumonia	42 (31.3)	7 (31.8)	35 (31.3)	0.958
OSAS	18 (13.4)	2 (9.1)	16 (14.3)	0.514
COPD	14 (10.4)	1 (4.5)	13 (11.6)	0.322
CHF	86 (64.2)	14 (63.6)	72 (64.3)	0.954
NYHA class, n (%)				0.564
I	11 (8.2)	1 (4.5)	10 (8.9)	
II	24 (17.9)	2 (9.1)	22 (19.6)	
III	25 (18.7)	5 (22.7)	20 (17.9)	
IV	9 (6.7)	2 (9.1)	7 (6.3)	
LVEF class, n (%)				0.888
Reduced	28 (20.9)	5 (22.7)	23 (20.5)	
Mildly reduced	10 (7.5)	2 (9.1)	8 (7.1)	
Preserved	86 (64.2)	13 (59.1)	73 (65.2)	
Albumin, g/dL	3.4 (3.0–3.8)	3.2 (2.4–3.3)	3.5 (3.1–3.8)	0.005
Creatinine, mg/dL	1.5 (1.1–2.7)	1.6 (0.9–2.1)	1.4 (1.1–2.7)	0.617
Sodium, mmol/L	136 (132–138)	135 (132–138)	136 (133–138)	0.520
CRP, mg/dL	3.5 (1.2–9.7)	5.8 (2.6–19.1)	3.1 (0.9–7.3)	0.041
Hemoglobin, g/dL	12.4 (10.2–14.5)	13.2 (10.5–14.8)	12.4 (10.1–14.4)	0.462
Hematocrit, %	38 (31.7–44.2)	39.5 (32.3–44.1)	37.5 (31.6–44.1)	0.599
BNP, pg/mL	758 (386–1,501)	785 (401–2,598)	748 (378–1,458)	0.357
Troponin, I ng/L	11.6 (0.1–49.9)	35.5 (0.1–82.6)	11.2 (0.1–37.3)	0.348

BMI: body mass index; CKD: chronic kidney disease; MASH: metabolic dysfunction-associated steatohepatitis; OSAS: obstructive sleep apnea syndrome; COPD: chronic obstructive pulmonary disease; CHF: chronic heart failure; NYHA: New York Heart Association; LVEY: left ventricular ejection fraction; CRP: C-reactive protein; BNP: brain natriuretic peptide. Values are expressed as medians (p25–p75) or frequencies (percentages).

During the follow-up period, 22 in-hospital deaths (16.4%) occurred. The median follow-up period was 9 days, and the overall hospital survival rate was 83.6%. The in-hospital mortality group presented a greater proportion of patients with liver cirrhosis and had higher Imp-R values (0.89 vs. 0.86, p=0.002) and lower body mass index (BMI) (23 vs. 27.4 kg/m^2^, p=0.011), abdominal SMA (86.5 vs. 111 cm^2^, p=0.024), pectoral SMA (18.9 vs. 26 cm^2^, p=0.005), PhA (3 vs. 3.9°, p=0.010), and serum albumin levels (3.2 vs. 3.5 g/dL, p=0.005) than the non-mortality group (Tables 1 and 2).

**Table 2:** Hydration and muscle mass assessment comparison in patients with in-hospital mortality.

Variables		Total (n=134)	Mortality (n=22)	Non-mortality (n=112)	p-value
Muscle assessment					
	Psoas SMA cm^2^	11.5 (8.8–14)	10.2 (6.9–11.8)	11.6 (9.12–14.9)	0.146
	Abdominal SMA cm^2^	105 (86.1–126)	86.5 (79.8–103)	111 (88.8–146)	0.024
	Abdominal SMI cm^2^/m^2^	42.8 (34.9–52)	35.3 (32.2–41.9)	45.8 (38.2–52.9)	0.015
	Low abdominal muscle mass n (%)	30 (22.4)	9 (40.9)	21 (18.75)	0.133
	Pectoralis SMA cm^2^	24.8 (19.9–33.8)	18.9 (16.3–26.2)	26 (21.1–34.7)	0.005
	Low pectoralis muscle mass n (%)	23 (17.2)	9 (40.9)	14 (12.5)	<0.001
Hydration status assessment					
	Third space water L	0.51 (-0.5 to 1.6)	0.55 (-1.25 to 1.88)	0.51 (-0.5 to 1.5)	0.614
	Impedance ratio	0.860 (0.840–0.883)	0.887 (0.861–0.898)	0.857 (0.834–0.880)	0.002
	Abnormal fluid distribution n (%)	83 (61.9)	19 (86.4)	64 (57.1)	0.016
	Phase angle°	3.7 (3–4.5)	3 (2.73–3.75)	3.9 (3.02–4.6)	0.010
Hydration state n (%)					0.383
	Overhydration	106 (79.1)	20 (90.9)	86 (76.8)	
	Normohydration	25 (18.7)	2 (9.1)	23 (20.5)	
	Dehydration	1 (0.7)	0	1 (0.9)	

SMA: Skeletal muscle area; SMI: skeletal muscle index. Values are expressed as medians (p25–p75) or frequencies (percentages).

In the multivariate logistic regression analysis, albumin, C-reactive protein (CRP), liver cirrhosis, abdominal SMI and SMA, low abdominal muscle mass, pectoral SMA, low pectoralis muscle mass, PhA, Imp-R, and abnormal fluid distribution were variables. The model with the better fit showed that low pectoralis SMA (OR 4.54, 95%CI 1.16–17.7, p=0.029), presence of liver cirrhosis (OR 9.88, 95%CI 1.23–79.15, p=0.031), and CRP (OR 1.09, 95%CI 1.01–1.18, p=0.025) were associated with in-hospital mortality.

Given the greater prevalence of low pectoralis muscle mass (40.9 vs. 12.5%, p<0.001) and increased Imp-R (86.4 vs. 57.1%, p=0.016) in the mortality group than in the non-mortality group, Kaplan-Meier survival curves were generated by classifying the patients into three groups: (1) normal pectoralis SMA with normal Imp-R, (2) normal pectoralis SMA with increased Imp-R, and (3) decreased pectoralis SMA with increased Imp-R. None of the patients had decreased pectoralis SMA or normal Imp-R; therefore, this group was excluded.

**Figure 2:**
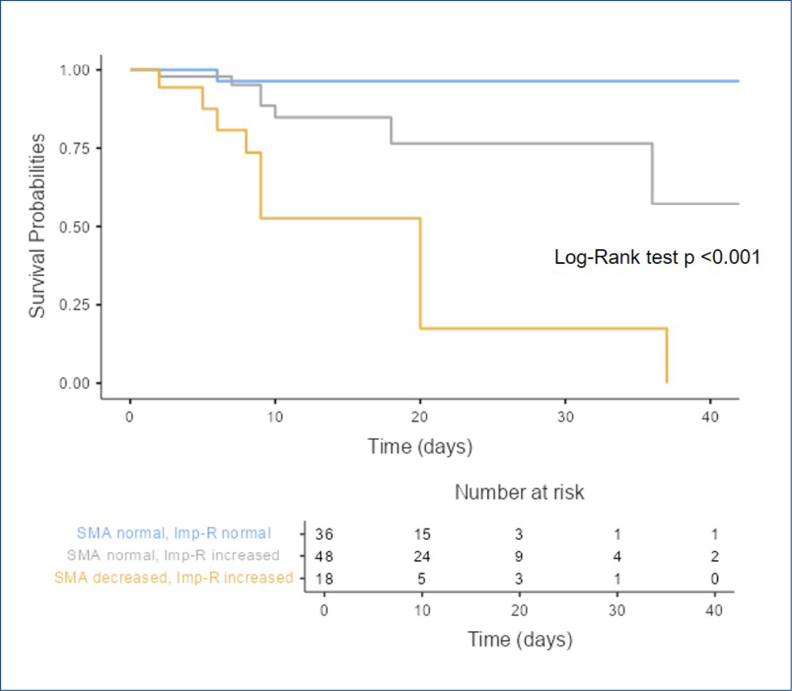
Kaplan-Meier survival of in-hospital mortality for pectoralis skeletal muscle mass and Imp-R. SMA: skeletal muscle mass; Imp-R: impedance ratio.

As shown in Figure 2, the group with decreased SMA and increased Imp-R levels had the lowest survival probability (log-rank test, p<0.001). This group also had the lowest hematocrit, hemoglobin, and albumin levels and the highest troponin I level compared to the other two groups.

## DISCUSSION

The main findings of this study were the presence of low muscle mass and fluid abnormalities, predominantly tissular congestion, which were associated with reduced survival during hospitalization for AHF.

CT is considered one of the best options for measuring muscle mass due to its availability in emergency settings and the ability to describe the overall condition of the centralized musculature. Chronic muscle wasting is more reliably assessed by evaluating the central appendicular muscles, which are more susceptible to change due to physical activity or hospital stay immobility^6,18^. Many cutoff points have been published for abdominal CT. However, as thoracic CT is more frequently performed than abdominal CT in patients with HF, pectoralis muscles are often a useful clinical indication, in addition to correlating with the abdominal SMA and being a good predictor of death in patients with HF^6^. The psoas and paraspinal muscles support the lumbar spine and femoral head, connect the trunk and lower extremities, and support an upright position while standing or sitting. The pectoralis muscles support movement between the trunk and arms, and their deterioration may contribute to the exacerbation of dyspnea, fatigue, and reduced cardiorespiratory fitness in HF^19^. Therefore, a reduction in these muscles may determine the clinical prognosis and mobility of patients with HF.

Physical inactivity and muscle wasting have been shown to increase peripheral vascular resistance and hypoperfusion, leading to endothelial dysfunction and sustained activation of compensatory mechanisms in HF^20^. Increased muscle catabolism is associated with low-grade chronic inflammation, reactive oxygen species circulation, insulin resistance, and fat mass accumulation, leading to cardiovascular alterations^20,21^. High levels of pro-inflammatory markers, such as interleukin 1 and 6, tumor necrosis factor, and CRP, promote AHF exacerbation and are elevated in patients with HF with sarcopenia and frailty^22^. Increased CRP levels were found in the mortality group in our study, which was consistent with the lower muscle mass and disease severity.

Regarding bioimpedance findings, a lower PhA was observed in the mortality group, which is a clinical predictor of disease severity and functional capacity, determined by central and peripheral congestion, and moderately by nutritional status in HF^23^. A high-frequency impedance (200 kHz) may pass through the cell membrane, describing total body water, whereas the low-frequency impedance (5 kHz) only reflects extracellular water; thus, Imp-R describes the proportion of total body water and extracellular water. Hence, a high value indicates a predominant fluid accumulation in the extracellular space, suggesting a tissue congestion phenotype^14^. Likewise, PhA and Imp-R describe cellular health and cellular membrane integrity^5,14^; therefore, the alteration of these markers, along with reduced serum albumin levels in the mortality group, could explain the fluid imbalance. The association between high Imp-R and low albumin levels and worsening renal function in decompensated HF has been described previously^24^.

Hypoalbuminemia and cellular damage may worsen owing to impaired muscle breakdown and wasting. Additionally, a lower plasma oncotic pressure, decreased interstitial compliance, or disruption of the endothelial glycocalyx can lead to fluid leakage into the interstitial space^11^. The differences between the congestion phenotypes within the groups may influence the response to treatment^1,4^. Therefore, assessing and characterizing congestion is essential for the clinical management of AHF^25^.

In addition, low muscle mass plays a crucial role in AHF prognosis; therefore, its assessment and treatment must be encouraged. Nutritional interventions (diet optimization, specific recommendations, and nutritional supplementation, if needed) along with physical activity (both aerobic and resistance exercises) are the main strategies^7^.

A limitation of this study is that although the use of CT images is a reliable and reproducible technique for measuring muscle mass, CT is expensive and accessible only in some health care centers, and it is not available for all patients with AHF. Additionally, metallic cardiac devices affect the visibility of the pectoralis muscles, limiting the use of thoracic CT in these patients. Thus, CT availability restricted the sample size in this study and the use of multivariate analysis. Furthermore, no cutoff values for SMA specific to this population are available. Consecutive measurements of additional parameters regarding treatment response were not considered in this study (such as BNP, creatinine, albumin, bioelectrical impedance vectorial analysis [BIVA], diuresis, and diuretic treatment), which may have improved the prognostic model.

Regarding the strengths of this study, we can mention that, to our knowledge, no other study has explored the relationship between Imp-R and pectoral SMA by CT with AHF survival, alone or in combination, and assessing changes in BIA parameters alongside SMA by CT during AHF hospitalization may provide valuable information about the usefulness of continuous monitoring of muscle mass and hydration state.

Future research should investigate the utility of these tools in optimizing decongestion and nutrition therapies, using a larger sample size. A key consideration is the assessment of global body composition and nutritional status, with emphasis on the preservation and recovery of muscle mass. This approach may improve HF compensation and patient outcomes. As multiple BIA parameters can identify distinct congestion phenotypes, their use offers a path toward more tailored and effective HF treatment.

## CONCLUSION

Moreover, measuring muscle mass using computed tomography is a useful tool that could help identify patients who may suffer from muscle wasting, which may influence disease progression. Bioimpedance provides diverse indicators that allow the identification of the congestion phenotype alongside other parameters. In conclusion, muscular loss and congestion were associated with survival during hospitalization for AHF.

## Data Availability

The datasets generated and/or analyzed during the current study are available from the corresponding author upon reasonable request.
