# Supplementary material for: Skeletal muscle mass and overhydration are associated with in-hospital mortality in acute heart failure patients
Source: Rev Assoc Med Bras (1992). 2026 May 1;72(2):e20250908. doi: 10.1590/1806-9282.20250908 (PMC13143153; doi:10.1590/1806-9282.20250908)
Supplement: Supplementary Table 1 [file 1806-9282-ramb-72-02-e20250908-suppl1.docx]

**Supplementary Table 1.** Intra-operator test–retest repeatability.

| Muscle | Right | 95%CI | Left | 95%CI | Summatory | 95%CI |
| --- | --- | --- | --- | --- | --- | --- |
| Psoas | 0.98 | 0.96–0.99 | 0.98 | 0.95–0.99 | 0.99 | 0.97–0.99 |
| Lumbar square | 0.92 | 0.82–0.97 | 0.77 | 0.53–0.90 | 0.9 | 0.77–0.96 |
| Paraspinal | 0.92 | 0.81–0.97 | 0.91 | 0.80–0.96 | 0.94 | 0.85–0.97 |
| Obliques | 0.96 | 0.91–0.98 | 0.93 | 0.84–0.97 | 0.98 | 0.94–0.99 |
| Rectus abdominis | 0.89 | 0.75–0.95 | 0.89 | 0.75–0.95 | 0.93 | 0.84–0.97 |
| Abdominal SMA | 0.99 | | | | | 0.97–0.99 |
| Pectoralis major | 0.98 | 0.96–0.99 | 0.98 | 0.95–0.99 | 0.99 | 0.97–0.99 |
| Pectoralis minor | 0.97 | 0.92–0.99 | 0.99 | 0.97–0.99 | 0.99 | 0.96–0.99 |
| Thoracic SMA | 0.99 | | | | | 0.98–0.99 |

CI: confidence interval; SMA: skeletal muscle area.
